# Long-term postoperative survival prediction in patients with colorectal liver metastasis

**DOI:** 10.18632/oncotarget.20322

**Published:** 2017-08-18

**Authors:** Kun Wang, Wei Liu, Xiao-Luan Yan, Juan Li, Bao-Cai Xing

**Affiliations:** ^1^ Hepatopancreatobiliary Surgery Department I, Key Laboratory of Carcinogenesis and Translational Research, Ministry of Education, Peking University School of Oncology, Beijing Cancer Hospital and Institute, Beijing, PR China

**Keywords:** colorectal cancer, KRAS mutation, colorectal liver metastasis, chemotherapy, survival

## Abstract

Numerous factors affect the prognosis of colorectal liver metastasis (CRLM) patients after hepatic resection. We investigated several factors related to overall survival in patients with CRLM to identify those most likely to benefit from hepatic resection, and produced a rational tumor biology score system. Three hundred CRLM patients treated with preoperative chemotherapy followed by hepatic resection between 2006 and 2016 were enrolled in our study. Clinicopathologic and long-term survival data were collected and assessed. Patient 1-, 3-, and 5-year overall survival rates were 92.7%, 58.3%, and 45.8%, respectively, while 1-, 3-, and 5-year disease-free survival rates were 44.7%, 28.6%, and 24.2%, respectively. Multivariate Cox regression analysis revealed poor preoperative chemotherapy response, Fong clinical risk score > 2, and KRAS mutation to be independent prognostic indicators in CRLM patients. As part of a preoperative staging system in which one point was assigned for each factor, a total score (out of 3) was predictive of long-term survival following surgery. These factors facilitate personalized prognostic assessments in CRLM patients planning for resection.

## INTRODUCTION

Approximately 50% of patients with primary colorectal cancer will develop liver metastases during their disease course [1]. Hepatic resection remains the only potentially curative treatment option for patients with colorectal liver metastasis (CRLM). While 5-year survival rates can be as high as 50–58%, patient prognoses can vary considerably [2, 3].

Criteria used to select CRLM patients for hepatic resection are based largely on clinical and radiologic parameters, such as tumor size and number, and response to preoperative chemotherapy, that attempt to predict prognosis post-resection [4–6]. Preoperative chemotherapy can shrink metastases and increase their resectability, and may also help select patients most likely to benefit from surgery [7, 8]. However, the prognostic landscape for predicting long-term outcomes in patients undergoing CRLM resection is changing [9]. Many prognostic models now rely on clinicopathological factors and tumor-specific molecular markers. Combining multiple factors within a single scoring system would better aid clinical decision-making. Thus, the present study investigated various tumor-related factors to develop a scoring system to predict CRLM patient survival following hepatic resection.

## RESULTS

This study analyzed 300 patients who received preoperative chemotherapy and underwent hepatic resection to treat CRLM between January 2006 and December 2016 (Table 1). No patients died from postoperative complications within 90 days of surgery, and all were eligible for the final analysis. Primary tumor resection was performed before hepatic resection in 173 patients (57.7%), during in 77 (25.7%) and after in 44 (16.6%). Liver metastasis was diagnosed synchronously in 265 patients (88.3%). Concomitant extrahepatic disease was present in 54 patients (18%). Conversion and neoadjvuant chemotherapy features are summarized in Table 2. Preoperative chemotherapy was administered to all patients with a median of four (range, 1–16) cycles. Major hepatic resection was performed in 135 patients (45.0%). 190 patients had a KRAS mutation, 155 had a Fong clinical risk score (CRS) > 2, and 182 exhibited poor response to preoperative chemotherapy.

**Table 1:** Demographic and clincial characteristics of study patients

Variable	No. of patients
Patients demographics	
Age (years)	55 (21–82)
Sex ration (M:F)	196:104
Primary T category	
T1–2	50 (16.7%)
T3–4	250 (83.3%)
Primary N category	
N0	136 (45.3%)
N1–2	164 (54.7%)
Primary tumor location	
Colon	128 (42.7%)
Rectum	172 (57.3%)
Primary tumor	
Right	53 (17.7%)
Left	247 (82.3%)
Primary tumor resection	
Before hepatectomy	179 (59.7%)
During hepatectomy	77 (25.7%)
After hepatectomy	44 (14.6%)
Timing of liver metastasis	
Synchronous	265 (88.3%)
Metachronous	35 (11.7%)
Tumor no.	3 (1–22)
Tumor size (mm)	25 (1–150)
Localization of liver metastases	
Unilobar	135 (45.0%)
Bilobar	165 (55.0%)
CEA level (ng/ml)	7.11 (0.1–861)
Concomitant extrahepatic disease	54 (18.0%)
Pre-treatment	
Conversion	65 (21.7%)
Neoadjuvant	235 (78.3%)

**Table 2:** Details of preoperative chemotherapy and hepatic resection

Variable	No. of patients
Chemotherapy before hepatic resection	
No. of cycles	4 (1–16)
No. of lines	
First line	237 (79.0%)
Second line	56 (18.7%)
Third line	7 (2.3%)
Response to last-line chemotherapy	
Complete	3 (1.0%)
Partial	128 (42.7%)
Stable disease	141 (47.0%)
Progressive disease	28 (9.3%)
Surgery details	
Operation time(min)	221.7 ± 84.0
Blood lose(ml)	272.0 ± 99.0
Hepatic resection	
Major resection	135 (45.0%)
Minor resection	165 (55.0%)
Margine status	
Positive	58 (19.3%)
Negative	242 (80.7%)
Complication	
Major(Clavien grade ≥ 3)	12 (4.0%)
Minor(Clavien grade < 3)	26 (8.7%)

### Long-term outcomes

**Figure 1:**
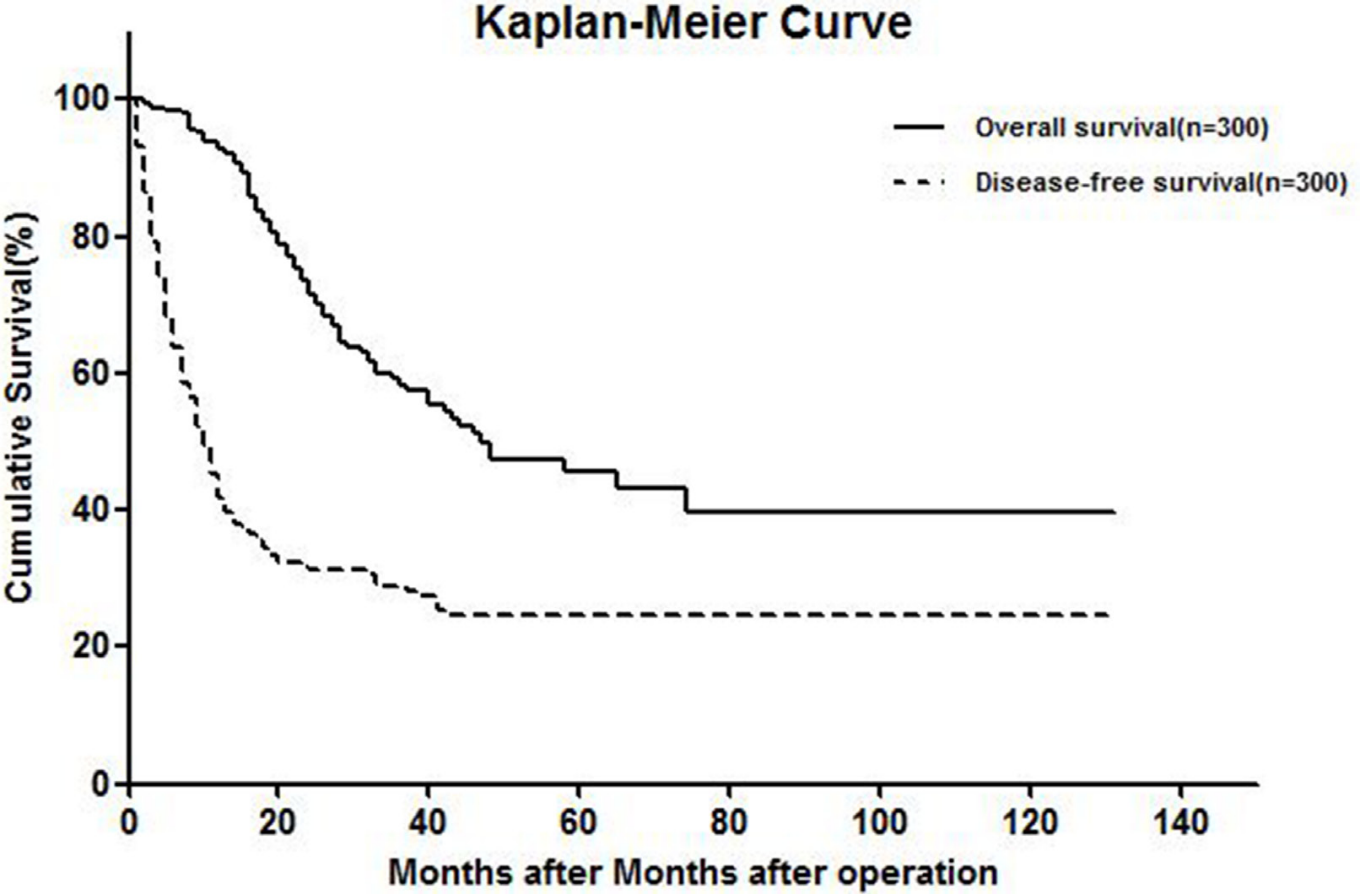
Kaplan-meier curve showing OS and DFS

Median follow-up time was 45 (range, 1–131) months, and no patients died during follow-up. Cumulative overall survival (OS) rates 1, 3, and 5 years after hepatic resection were 92.7%, 58.3%, and 45.8%, respectively. Cumulative disease-free survival (DFS) rates 1, 3, and 5 years after surgery were 44.7%, 28.6%, and 24.2% respectively, on an intention-to-treat basis (Figure 1).

### Univariate and multivariate analyses of factors associated with OS

In univariate analyses, five variables, including KRAS mutation, Fong CRS > 2, tumor number>1, size > 5 cm at diagnosis, and poor response to preoperative chemotherapy, were associated with decreased OS (*p <* 0.050) (Table 3). These variables were included in a subsequent multivariate Cox regression analysis, which identified three independent prognostic factors for OS: Fong CRS > 2 (HR: 4.247; 95% CI: 1.758–8.260; *p* = 0.001), KRAS mutation (HR: 2.196; 95% CI: 1.372–3.515; *p <* 0.001) and poor response to preoperative chemotherapy (HR: 2.054; 95% CI: 1.025–4.119; *p* = 0.042) (Table 3).

**Table 3:** Prognostic factors of overall survival after hepatectomy

Analysis		n	5-year OS	HR		95% CI	*p* value
**Univaraite**							
Age	> 60 y	94	40.7%	0.874		0.556–1.375	0.561
	≤ 60	206	47.6%				
Sex	Male	196	39.9%	1.215		0.773–1.911	0.399
	Female	104	56.4%				
Primary T category	T1-T2	50	40.9%	0.849		0.494–1.459	0.554
	T3-T4	250	46.7%				
Primary N category	N0	136	44.7%	1.079		0.711–1.638	0.721
	N1	164	45.6%				
Primary tumor location	Left	247	42.4%	1.325		0.735–2.368	0.349
	Right	53	42.0%				
Timing of liver metastasis	Synchronous	265	43.8%	1.497		0.748–2.995	0.255
	Metchronous	35	58.5%				
Tumor no. at diagnosis	*n =* 1	80	54.5%	1.686		1.012–2.809	0.045
	*n* > 1	220	42.7%				
Tumor size at diagnosis	> 50 mm	41	22.7%	2.414		1.518–3.863	0.000
	≤ 50	259	53.2%				
Location of liver metastasis	Bilobar	165	43.9%	1.262		0.826–1.928	0.282
	Unilobar	135	46.8%				
CEA level(ng/ml)	> 200	15	46.4%	2.114		0.917–4.877	0.079
	≤ 200	285	46.7%				
Concomitant extrahepatic disease	Yes	54	41.3%	1.408		0.896–2.281	0.164
	No	246	47.2%				
Major hepatectomy	Yes	135	40.2%	1.436		0.947–2.177	0.089
	No	165	50.3%				
CRS	≤ 2	155	57.5%	2.427		1.575–3.740	0.000
	> 2	145	32.6%				
KRAS status type	Wild	190	53.6%	2.691		1.772–4.086	0.000
	Mutation	110	31.7%				
Worse chemotherapy response	Yes	182	33.8%	2.010		1.322–3.057	0.001
	No	118	51.8%				
Margin	Positive	58	45.1%	1.029		0.491–2.156	0.939
	Negative	242	46.3%				
Pre-treatment	Neo	235	48.2%	1.130		0.679–1.881	0.637
	Con	65	33.1%				
Primary tumor	Rectum	172	50.1%	1.301		0.859–1.972	0.214
	Colon	128	39.9%				
Mjaor complications	Yes	12	42.8%	1.228		0.674–2.236	0.117
	No	288	47.6%				
**Multivaraite**							
Tumor no. at diagnosis	*n* > 1	220			2.588	0.787–8.511	0.117
Tumor size at diagnosis	> 50 mm	41			3.140	0.935–4.542	0.064
CRS	> 2	155			4.247	1.758–8.260	0.001
KRAS status	Mutation	190			2.196	1.372–3.515	0.001
Chemotherapy response	Worse	182			2.054	1.025–4.119	0.042

### Tumor biology score

**Figure 2:**
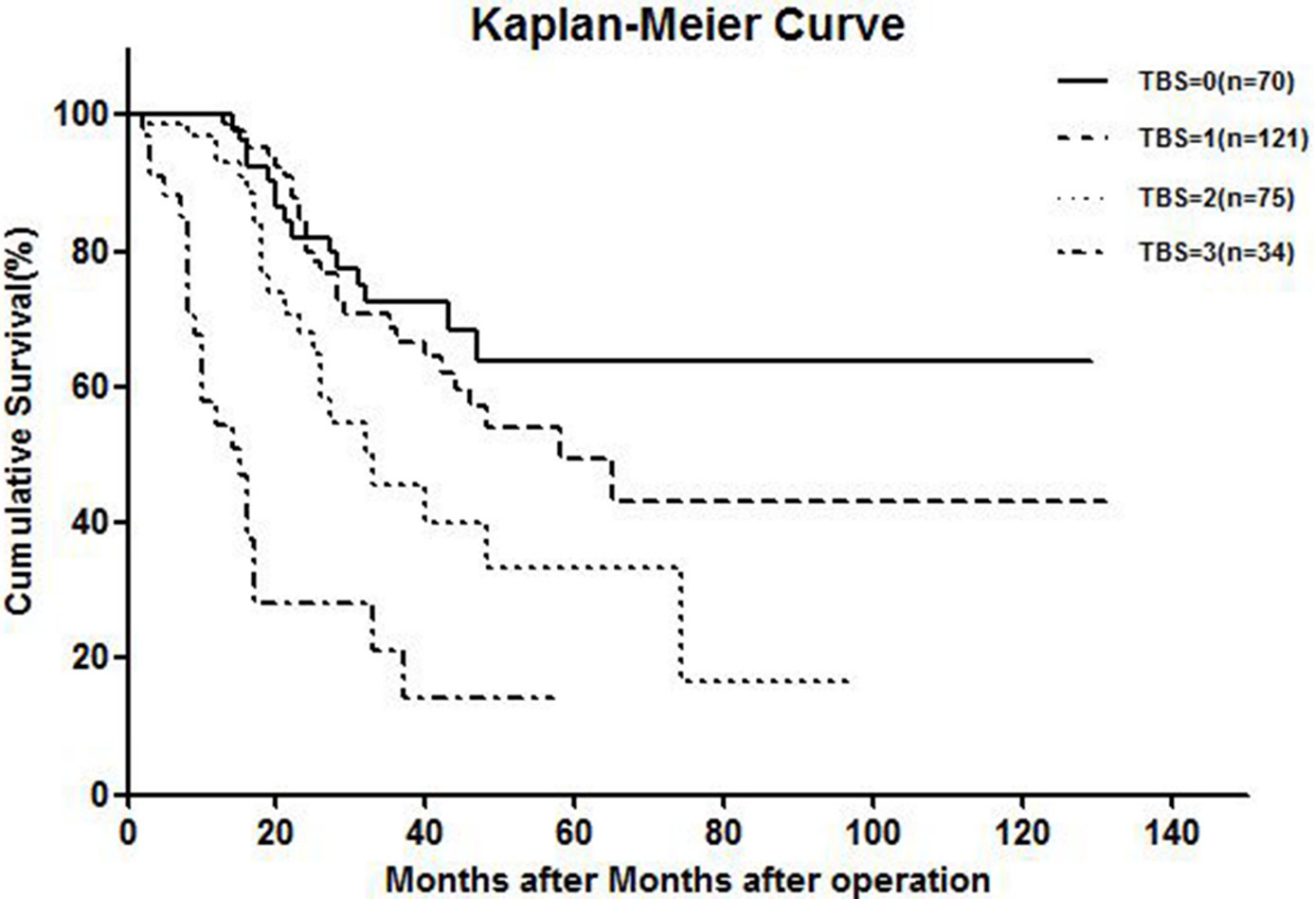
Kaplan-meier curve showing overall survival of TBS system

KRAS mutation, Fong CRS > 2, and poor preoperative chemotherapy response were chosen as criteria for a tumor biology score (TBS) staging system. Each risk factor was assigned one point, and total score was compared with the clinical outcome of each patient after hepatic resection. Five-year OS rates for patients scoring TBS 0, 1, 2, and 3 were 63.7%, 49.6%, 33.3%, and 14.1%, respectively (Figure 2).

### Prognostic predictive power

Nine eligible studies satisfied our inclusion criteria, and patient demographic and clinicopathological data were extracted (Supplementary Table 1) [4, 5, 10–16]. The predictive powers of the Fong, Iwatsuki, Konopke, Nagashima, Nordlinger, Pawlik, Rees, Vauthey scores, and our TBS were 0.585 (95% CI: 0.520–0.549; *p =* 0.011), 0.513 (95% CI: 0.443–0.584; *p* = 0.710), 0.585 (95% CI: 0.474–0.696; *p* = 0.098), 0.509 (95% CI: 0.443–0.575; *p* = 0.788), 0.529 (95% CI: 0.464–0.594; *p* = 0.385), 0.524 (95% CI: 0.451–0.598; *p* = 0.506), 0.510 (95% CI: 0.440–0.581; *p* = 0.769), 0.615 (95% CI: 0.531–0.699; *p* = 0.043), and 0.642 (95% CI: 0.570–0.713; *p* = 0.036), respectively.

## DISCUSSION

This study investigated several prognostic factors related to OS in patients with CRLM, and produced a rational tumor biology score system. Multivariate Cox regression analysis identified three variables, including KRAS mutation, Fong CRS > 2, and poor preoperative chemotherapy response, as independent prognostic factors for CRLM patients who plan to undergo hepatic resection.

In selected patients with unresectable disease, conversion chemotherapy may allow for secondary resection and improved long-term survival [17]. Neoadjuvant chemotherapy has been also proposed for patients with resectable disease and negative prognostic factors to better select those who could benefit from liver surgery, and to avoid surgery in patients with rapidly progressing tumors [18]. RECIST was established to assess cytotoxic treatment effects in solid tumors, and CRLM response to preoperative chemotherapy has prognostic value [8]. Partial response suggests a better prognosis, while stable disease is likely due to tumor cell resistance. However, recent findings question the effectiveness of RECIST in colorectal cancer patients [19]. The conventional tumor size-based radiologic criteria of RECIST may be inadequate in assessing response to chemotherapy, especially in patients treated with a regimen including bevacizumab [20]. Lesions that are predominantly necrotic may not be ideal RECIST targets, because their attenuation closely mimics that of a treated lesion. In some instances, stable disease response may be inconsistent with improvement via RECIST criteria, but still associated with an optimal morphologic response [21]. More recently, investigators have proposed pathological and radiological responses to chemotherapy as alternative outcome endpoints for predicting survival after CRLM resection [21, 22]. However, pathological response can be assessed only after surgery, and survival was associated with radiological response primarily in patients receiving preoperative anti-vascular endothelial growth factor (VEGF) therapy [23, 24].

Several clinical risk scores (CRS) have been developed to predict tumor recurrence and survival after CRLM resection [4, 5]. The most validated and widely-used CRS was described by Fong, *et al.* in 1999 [4]. The Fong CRS identified five independent prognostic clinical variables predicting survival after CRLM surgery, and characterized two risk groups: patients with a high-risk profile have worse OS rates than low-risk patients. Although all CRLMs may be considered high risk, this CRS may explain the relative lack of systemic therapy efficacy when combined with surgery in the metastasized setting. Tomlinson, *et al.* demonstrated in CRLM surgery 10-year sur*vivo*rs that patients with a low Fong CRS had a cure rate of 21% versus 10% in patients with a high CRS [25]. In high CRS patients, perioperative chemotherapy was associated with a survival advantage, but was of no benefit in low CRS patients. Data suggests that patients with a low CRS have a favorable tumor biology [26]. However, large, single-institution studies have questioned the validity and clinical usefulness of risk scores [27, 28]. The prognostic significance of most of these factors was determined at a time when effective cytotoxic agents were not available. Consequently, although most of these factors are still routinely used, their utility as prognostic indicators in the era of modern chemotherapy is uncertain and should be reassessed.

While many prognostic models now rely on clinicopathological factors, molecular biomarkers are likely to replace traditional clinical and morphometric factors [9, 29]. KRAS mutations are associated with tumor cell migration and invasion via disruption of the actin cytoskeleton and regulation of integrin expression, among other mechanisms [30, 31]. The prognostic importance of activating KRAS mutations extends beyond predicting sensitivity to anti-EGFR monoclonal antibodies, and may reflect a more migratory and invasive tumor biology resulting in early and frequent recurrences after hepatic resection. Historically, survival predictions were based on primary tumor and metastases morphological characteristics. Factors associated with aggressive or advanced tumor biology, such as bilobar disease, multiple metastases, large metastases, and metastases in difficult locations, are also associated with technically complex cases.

In conclusion, our analysis revealed three important factors for predicting prognosis in CRLM patients undergoing surgery. Characterization of novel biomarkers in these patients will enhance our understanding of CRLM aggressiveness, assist in clinical decision-making, and help to identify new, more efficient therapies.

## MATERIALS AND METHODS

### Patients

Between January 2006 and December 2016, 300 CRLM patients received preoperative chemotherapy and underwent hepatic resection at the Hepatopancreatobiliary Surgery Department I of Peking University Cancer Hospital. CRLM diagnoses were all confirmed by histopathology.

### Preoperative management

As a result of the long study period, response to chemotherapy was classified according to World Health Organization criteria, which are in agreement with the Response Evaluation Criteria in Solid Tumors (RECIST) [32]. Poor preoperative chemotherapy response was represented by progressive disease or stable disease with the target lesion increased in diameter by < 30%. If the disease was not controlled with chemotherapy, systemic chemotherapy was restarted using another regimen. Response was then re-evaluated to assess the possibility of surgery. Response was based on the last-line preoperative chemotherapy before hepatic resection. Two radiologists reviewed all images from the 300 patients independently.

### Surgical treatment

The objective of surgery was to resect all detectable lesions with tumor-free margins. If obtaining a tumor-free margin was not possible due to contact with major vascular or biliary structures, resection was still indicated provided that all tumors could be resected macroscopically. All patients underwent hepatic resection with curative intent, and to achieve complete resection (R0) while preserving as much normal functional liver parenchyma (with adequate vascular inflow, outflow, and biliary drainage) as possible. Resection of three or more segments was considered a major hepatic resection. The normal liver parenchyma remnant volume was > 30%. For chemotherapy liver injury patients, the remnant volume should be preserved at > 40%. The presence of extrahepatic tumors was not considered a contraindication to hepatic resection if the lesions were limited and resectable. Extrahepatic disease identified in the abdominal cavity was resected at the same time as hepatic resection. For extrahepatic disease located outside the abdomen, resection was performed 2–3 months after hepatectomy if the disease remained controlled with interval chemotherapy.

### Postoperative treatment

Postoperative chemotherapy was recommended routinely, using the same protocol as that applied before surgery. Recurrence was treated surgically only when the overall strategy was considered potentially curative. All patients were followed up every three months for the first two years, with a physical examination, carcinoembryonic antigen (CEA) measurement, and abdominal ultrasonography. Every six months, patients underwent computed tomography scan of the abdominal/thoracic/pelvic region (enhanced MRI could replace CT). No patients died during follow-up.

### Statistical analysis

Continuous variables were summarized as means and categorical variables were summarized as frequencies and percentages. Qualitative variable comparisons were performed using Pearson’s chi-square test. Kaplan-Meier survival curves were calculated from the date of hepatic resection and differences were determined using a log-rank test. Clinicopathologic factors were analyzed using Cox’s proportional hazard model to identify independent risk factors for overall survival. *p* < 0.05 (two-sided) was considered significant. Statistical analyses were performed using SPSS 19.0 (SPSS, Inc., Chicago, IL, USA).

### Comprehensive MEDLINE review

We comprehensively searched the MEDLINE database using the following medical subject headings (MeSH): “colorectal liver metastases” or “colorectal metastasis” and “liver resection” or “hepatic resection” or “surgery”. We also manually searched relevant references and review articles. Studies were included in our review if they (a) proposed a scoring system to predict CRLM survival outcome, (b) were published in English, and (c) were published between January 1990 and April 2017, to ensure comparability with our retrospective clinical study. Studies involving fewer than 50 patients were excluded. To compare staging systems as predictors of prognosis after hepatic resection, we used the concordance c-statistic (as the area under the receiver operating characteristic [ROC] curve). Prognostic system performance is related to homogeneity (small differences in survival among patients at the same stage within each system), discrimination ability (greater differences in survival among patients at different stages within each system), and monotonicity of gradients (survival of patients at earlier stages is longer than that of patients at more advanced stages within the same system).

## SUPPLEMENTARY MATERIALS TABLES




