# Supplementary material for: Long-term postoperative survival prediction in patients with colorectal liver metastasis
Source: Oncotarget. 2017 Aug 18;8(45):79927–34. doi: 10.18632/oncotarget.20322 (PMC5668107; doi:10.18632/oncotarget.20322)
Supplement: Supplementary file 1 [file oncotarget-08-79927-s001.pdf]

Long-term postoperative survival prediction in patients with colorectal liver metastasis

SUPPLEMENTARY MATERIALS

Supplementary Table 1: Clinical risk score summary. See Supplementary_Table_1
