# Supplementary material for: Long-term postoperative survival prediction in patients with colorectal liver metastasis
Source: Oncotarget. 2017 Aug 18;8(45):79927–34. doi: 10.18632/oncotarget.20322 (PMC5668107; doi:10.18632/oncotarget.20322)
Supplement: Supplementary file 2 [file oncotarget-08-79927-s002.docx]

**Supplementary Table 1: Clinical risk score summary**

| **Scale** | **Year** | **Population** | **Region** | **Criteria** | **Score** |  |
| --- | --- | --- | --- | --- | --- | --- |
| Adam[10] | 1990-2012 | 439 | France | Lymph node–positive primary tumor | 0-16 | Low risk |
|  |  |  |  | Number of hepatic metastases>6 | >16 | High risk |
|  |  |  |  | CA199 level(Units/ml)>37 |  |  |
|  |  |  |  | Disease progression during first-line chemotherapy |  |  |
|  |  |  |  | Presence of extrahepatic disease |  |  |
| Fong[4] | 1985-1998 | 1001 | America | Number of liver metastases>1 | 0-2 | risk factors |
|  |  |  |  | Preoperative CEA level>200 ng/ml | 3-5 | risk factors |
|  |  |  |  | Largest size of liver metastasis≥5 |  |  |
|  |  |  |  | Lymph node–positive primary tumor |  |  |
|  |  |  |  | Interval less than 12months from primary tumor to metastases |  |  |
| Iwatsuki[11] | 1981-1996 | 305 | America | Number of hepatic metastases>2 | 0-1 | low risk |
|  |  |  |  | Largest size of liver metastasis≥8cm | 2-3 | intermediate risk |
|  |  |  |  | Lesion located in bilobar | >3 | high risk |
|  |  |  |  | Interval less than 30 months from primary tumor to metastases |  |  |
| Konopke[12] | 1993-2006 | 265 | Germany | Number of liver metastases≥4 | 0 | low risk |
|  |  |  |  | CEA level (ng/ml)≥200 | 1 | intermediate risk |
|  |  |  |  | Synchronous liver metastases | >1 | high risk |
| Nagashima[13] | 1981-1997 | 83 | Japan | Serosal invasion of primary tumor | 0-1 | grade 1 |
|  |  |  |  | Positive lymph node of primary tumor | 2-3 | grade 2 |
|  |  |  |  | Number of hepatic metastases≥2 | >3 | grade 3 |
|  |  |  |  | Largest size of liver metastasis≥5 |  |  |
|  |  |  |  | Resectable extrahepatic metastases |  |  |

**Supplement Table Clinical risk score summary**

| **Scale** | **Year** | **Population** | **Region** | **Criteria** | **Score** |  |
| --- | --- | --- | --- | --- | --- | --- |
| Nordlinger[5] | 1968-1990 | 1568 | France | Age≥60 years | 0-2 | risk factors |
|  |  |  |  | Extension into the serosa of the primary cancer | 3-4 | risk factors |
|  |  |  |  | Lymphatic spread of the primary cancer | 5-6 | risk factors |
|  |  |  |  | Interval less than 2 years from primary tumor to metastases |  |  |
|  |  |  |  | Number of metastases≥4 |  |  |
| Pawlik[14] | 2000-2015 | 604 | America | TBS^2^=Diameter^2^+Number^2^ | <3 | grade 1 |
|  |  |  |  |  | 3-8 | grade 2 |
|  |  |  |  |  | ≥9 | grade 3 |
| Rees[15] | 1987-2005 | 929 | United Kingdom | Number of hepatic metastases>3 | 1-5 | low risk |
|  |  |  |  | Lymph node–positive primary tumor | 6-10 | intermediate risk |
|  |  |  |  | Poorly differentiated primary | 11-15 | high risk |
|  |  |  |  | Extrahepatic disease | >15 | very high risk |
|  |  |  |  | Largest size of liver metastasis>5 cm |  |  |
|  |  |  |  | Carcinoembyonic antigen level>60 ng/mL |  |  |
| Vauthey[16] | 2005-2013 | 564 | America | Lymph node–positive primary tumor | 0 | grade 1 |
|  |  |  |  | Largest size of liver metastasis>5 cm | 1 | grade 2 |
|  |  |  |  | RAS mutation | 2 | grade 3 |
|  |  |  |  |  | 3 | grade 4 |
